# Utilisation of Sulphur By-Products in Diverse Civil Engineering Applications: A Systematic Review

**DOI:** 10.3390/ma19040784

**Published:** 2026-02-18

**Authors:** Mohsin Usman Qureshi, Ali Al-Shamakhi, Mohammed Rumhi, Muhammad Ashraf Javid, Wan Hamidon Wan Badaruzzaman, Ghassan Al-Kindi, Wadhah M. Tawfeeq, Rakesh Belwal, Hajir Al-Handasi

**Affiliations:** 1 Faculty of Engineering, Sohar University, P.O. Box 44, Sohar 311, Oman; mjavid@su.edu.om (M.A.J.); whamidon@su.edu.om (W.H.W.B.); gkindi@su.edu.om (G.A.-K.); wtawfeeq@su.edu.om (W.M.T.); hhandasi@su.edu.om (H.A.-H.); 2 Petroleum Development Oman, P.O. Box 81, Muscat 100, Oman; ali.as.shamakhi@pdo.co.om (A.A.-S.); mohammed.mr.rumhi@pdo.co.om (M.R.); 3 Faculty of Business, Sohar University, P.O. Box 44, Sohar 311, Oman; rbelwal@su.edu.om

**Keywords:** sulphur, concrete, asphalt, durability, strength, PRISMA, bibliometric

## Abstract

**Highlights:**

**What are the main findings?**

- Research on sulphur application in civil materials has grown significantly.
- Sulphur concrete and sulphur asphalt exhibit durability, strength, and recyclability.
- Modified sulphur systems help overcome brittleness and temperature sensitivity.

**What are the implications of the main findings?**

- Sulphur waste streams can support sustainable construction and a circular economy.
- Sulphur materials offer techno-economic and environmental benefits for industry.
- Future work may validate in situ applications of sulphur concrete.

**Abstract:**

Sulphur, a major by-product of the oil and gas industry, has emerged as a promising construction material in both sulphur concrete (SC) and sulphur-extended asphalt (SEA) applications. This review examines the development, properties, and uses of these sulphur-based construction materials over a century by following PRISMA guidelines for systematic literature selection. A bibliometric analysis highlights a surge in research activity over the last two decades. The key advantages of sulphur concrete include rapid strength gain (achieving ~50 MPa within 1–2 days) and exceptional chemical durability in extreme environments. Sulphur-bound materials exhibit high corrosion resistance, low water permeability, and full recyclability upon reheating. Challenges such as thermal shrinkage-induced brittleness and temperature sensitivity have been mitigated by using polymer-modified sulphur and mix design optimisation. Sulphur-extended asphalts benefit from increased stiffness, stability, and cost savings compared to conventional mixtures. Enhanced performance has been observed at sulphur replacement levels of 20–40% in asphalt binders. The review also summarises mixed formulations, mechanical properties, durability metrics, and innovative applications ranging from acid-resistant industrial structures to sustainable pavement materials and even extraterrestrial construction. The environmental benefits, such as up to 40% GHG reduction and complete recyclability of sulphur-based concretes, align with circular economy goals. Future research directions include improving ductility, advancing 3D printing techniques, and field validation of long-term performance. Overall, sulphur by-products can be transformed into valuable construction materials that address waste management and infrastructure durability.

## 1. Introduction

**Figure 1:**
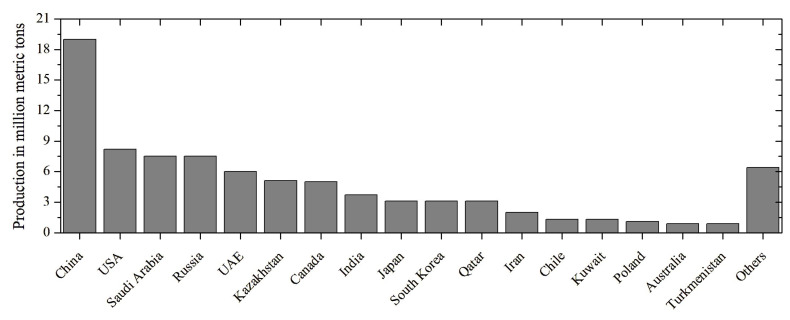
Worldwide sulphur production in 2024 [1].

The global desulphurisation of fossil fuels has resulted in a persistent oversupply of elemental sulphur, creating both an environmental management challenge and an opportunity for sustainable material innovation. As illustrated in Figure 1, the current global sulphur surplus has once again intensified interest in sustainable utilisation pathways. Among potential large-scale applications, construction materials offer a particularly attractive outlet due to their volume demand, durability requirements, and long service life. Sulphur concrete (SC), also referred to as elemental sulphur concrete, is produced by mixing molten sulphur (typically 130–140 °C) with dry aggregates, forming a cement-free composite that gains strength upon cooling rather than hydration. In this system, sulphur fully replaces Portland cement and water as the binder, eliminating curing requirements and enabling rapid strength development.

Early investigations into sulphur-based binders demonstrated exceptional chemical resistance but were limited by shrinkage cracking associated with sulphur’s phase transformation near 95 °C [2]. Renewed research since the 1970s addressed these limitations through optimised aggregate gradation and polymer modification, leading to modern sulphur concrete systems with improved thermal stability and durability [3,4]. Sulphur concrete exhibits several performance advantages over conventional Portland cement concrete, most notably rapid strength gain and superior resistance to aggressive chemical environments. Typical sulphur concretes achieve 40–60 MPa compressive strength within 1–2 days, reaching approximately 90% of ultimate strength within 24 h, compared with 28 days for conventional concrete [5,6,7,8,9,10,11,12,13]. The absence of water in mixing and curing enables placement in freezing or arid environments, while the dense, impermeable sulphur matrix yields extremely low water absorption and excellent resistance to acids, salts, and sulphates [14,15,16,17,18,19,20,21,22,23].

From a sustainability perspective, sulphur-based binders offer significant environmental benefits by offsetting Portland cement production, reducing CO_2_ emissions by at least 40%, and enabling fully recyclable, closed-loop construction systems [24,25,26,27,28,29]. At the same time, challenges related to brittleness, thermal sensitivity, and standardisation remain, motivating continued research into polymer-modified sulphur systems, optimised mix design, and field-scale validation [30,31,32,33,34,35,36,37,38,39]. Beyond rigid concrete applications, elemental sulphur has also been successfully incorporated into flexible pavements as sulphur-extended asphalt, partially replacing bitumen and improving stiffness, rutting resistance, and cost efficiency [24,26,31,38,40,41,42,43,44,45,46,47,48]. This review synthesises the state of the art in sulphur-based concrete and asphalt, critically examining material formulations, production technologies, mechanical and durability performance, sustainability implications, and emerging applications to identify pathways towards wider industrial adoption.

A systematic literature review methodology was adopted for this study. This approach focuses on gathering evidence on a specific topic, critically assessing and synthesising the available research, and identifying existing knowledge gaps that require further investigation in future work [49]. For a rigorous systematic review, the PRISMA guidelines (Preferred Reporting Items for Systematic Reviews and Meta-Analyses) were followed to ensure transparency, accuracy, and completeness in the selection, evaluation, and reporting of the literature. The PRISMA statement, first released in July 2009, was developed to help authors enhance the quality and clarity of systematic review reporting. It outlines the essential elements needed to accurately prepare, interpret, and apply the findings of a systematic review. PRISMA serves as a comprehensive set of guidelines for authors preparing review manuscripts [50,51,52]. The systematic review followed a structured strategy that identified the central industrial by-product of interest (sulphur), the intervention being examined (its utilisation in civil engineering applications), and the anticipated outcomes (practical industrial uses). Based on this framework, the key research questions were formulated for investigation.

1. How is sulphur currently used in civil engineering materials and applications?
2. What mechanical and durability properties do sulphur-based materials exhibit compared with conventional materials?
3. What environmental and economic benefits arise from using sulphur in construction?
4. What challenges and research gaps must be addressed to enable the wider industrial adoption of sulphur-based materials?

## 2. Methodology

The research followed a structured process, i.e., narrowing the topic, defining keywords, selecting databases, filtering irrelevant studies, and analysing the final literature set [53]. Quality assessment followed the PRISMA checklist 2020 (see the Supplementary Materials).

The eligibility criteria for the included studies are laboratory or field-based investigations with clearly defined conventional concrete or asphalt control mixes, the standardised incorporation of sulphur by-products, and comparable baseline material properties to ensure valid comparisons. Studies were also required to evaluate performance using well-defined mechanical and durability indicators, such as strength, stiffness, rutting resistance, moisture damage, and chemical resistance.

The exclusion criteria included studies unrelated to civil engineering applications, laboratory-scale chemical or material synthesis studies without structural relevance, narrative review articles, conference abstracts, and case reports.

Population, Intervention, Comparison, Outcomes (PICO):

Population: Civil engineering materials and systems, including concrete and asphalt mixtures used in structural, pavement, or infrastructure applications.

Intervention: Utilisation of sulphur by-products (e.g., elemental sulphur, modified sulphur, sulphur-based binders) as partial or full replacements in concrete or asphalt mixtures.

Comparison: Conventional concrete or asphalt mixtures without sulphur incorporation, or mixtures using traditional binders and aggregates.

Outcomes: Primary outcomes included mechanical performance and durability indicators such as compressive strength, tensile strength, stiffness, rutting resistance, moisture damage, and chemical or thermal resistance. Secondary outcomes included constructability, field performance, long-term behaviour, and practical implementation in civil engineering applications.

**Figure 2:**
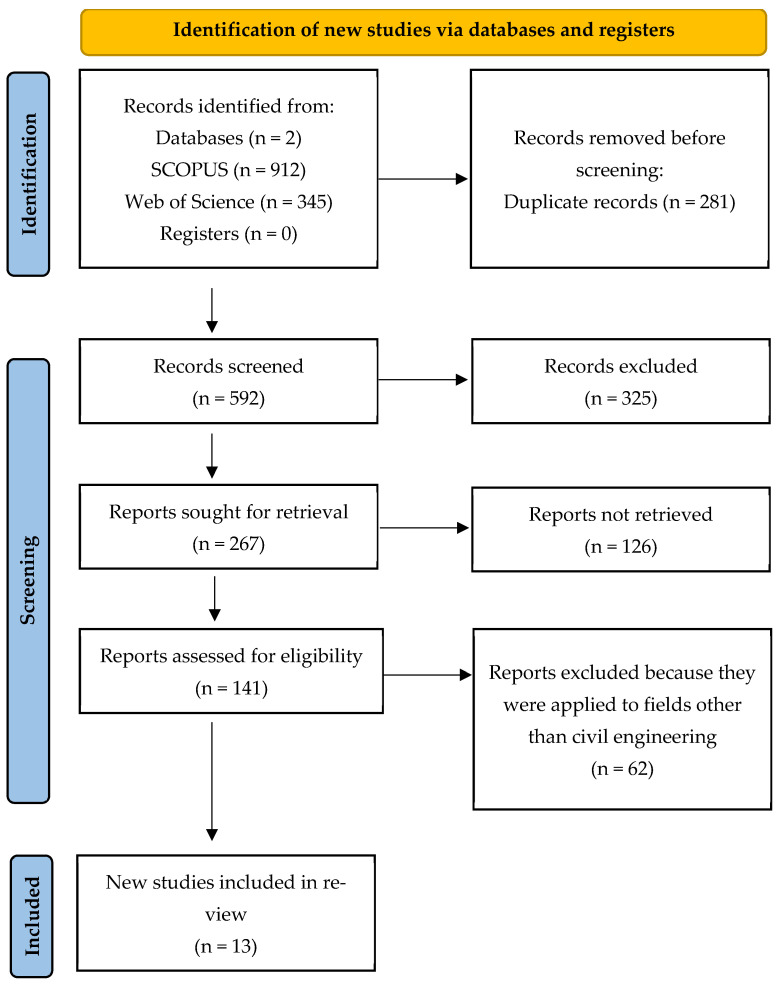
Selection of relevant articles for the systematic review on the use of sulphur by-products in civil engineering applications. PRISMA methodology flowchart [54].

The comprehensive electronic search using the keywords sulphur, sulfur, concrete, asphalt, and waste was performed in September 2025 across Scopus and Web of Science, applying Boolean operators as appropriate and without any time restriction. The initial search yielded 912 records, which were screened to remove duplicates and studies outside the research scope. The keyword combinations that yielded the most relevant results across the databases were (TITLE-ABS-KEY (sulphur) AND TITLE-ABS-KEY (sulfur) AND TITLE-ABS-KEY (concrete) AND TITLE-ABS-KEY (asphalt) AND TITLE-ABS-KEY (waste)). The detailed PRISMA-based search steps and exclusion criteria are presented in Figure 2.

Two independent reviewers screened titles and abstracts for relevance, followed by a full-text assessment of potentially eligible studies based on the predefined inclusion and exclusion criteria. Any discrepancies were resolved through discussion and consensus. All records were reviewed, and duplicate entries were removed. Manual screening of reference lists from the included studies and relevant reviews was also performed. Studies not related to civil engineering materials, material synthesis research without structural relevance, review articles, conference abstracts, and case reports were excluded. In contrast, studies addressing the utilisation of sulphur by-products in concrete or asphalt for civil engineering applications and reporting strength, durability, or field performance outcomes were included.

Owing to substantial heterogeneity in experimental methodologies, performance indicators, and sulphur incorporation approaches, a quantitative meta-analysis was not feasible. Instead, the findings were synthesised descriptively, focusing on comparative performance trends, consistency of observed effects, and the relative strength and durability behaviour of different sulphur-based material systems.

The present study also utilised scientometrics, a quantitative analytical tool that has become widely used for evaluating scientific output, tracking research progress, identifying emerging trends, and uncovering the underlying structure of knowledge domains across science, scientific communication, and science policy [51]. To contextualise the development of sulphur-based construction materials, a bibliometric analysis employing the bibloshiny package in R was conducted on the scientific literature from the 1970s to 2025 [52]. Using the above-stated tool, the study examined annual publication trends, thematic areas, and keywords related to sulphur by-product utilisation in construction, providing insight into both global and regional scales. Core keywords were identified, and co-occurrence networks and visual knowledge maps were developed to illustrate the distribution of research activity and the emergence of key thematic clusters.

## 3. Discussion on Concrete and Asphalt Applications

### 3.1. Materials and Processing

Sulphur concrete uses elemental sulphur (S_8_) as the binder, typically a by-product of oil and gas desulphurisation. Sulphur is heated above its melting point (~119 °C) to form a low-viscosity liquid. At temperatures above ~159 °C, polymerisation and hydrogen sulphide (H_2_S) release may occur, requiring careful control. Mixing is therefore conducted at 130–145 °C to ensure workability and safety [29,55]. ACI recommends 132–141 °C during handling [11], while asphalt applications use ~140 °C, kept below ~155 °C [47].

Modified sulphur binders improve performance. Dicyclopentadiene (DCPD), typically added at 5–10%, reacts with sulphur to form polymeric sulphur cement, reducing crystallinity and shrinkage [56]. Organic polysulfide polymers (Thiokol) enhance grout stability. In sulphur-extended asphalt, additives such as Shell’s Thiopave suppress fumes and improve mixing [33]. Bitumen-modified sulphur concrete (BMSC) incorporates 5–10% bitumen to increase ductility and reduce cracking [6,57].

Sulphur concrete uses conventional aggregates such as sand, gravel, or crushed stone, which must be completely dried and often preheated up to 150 °C to avoid foaming or explosions when in contact with molten sulphur. Well-graded aggregates are preferred to minimise voids, binder demand, shrinkage, and cost [58]. Fine fillers (5–15%), including fly ash, limestone dust, or silica flour, improve packing density and suspension stability [59]. In road applications, dense-graded limestone and sand aggregates have shown strengths comparable to cement concrete [45].

Typical sulphur concrete contains 15–30% sulphur binder and 70–85% aggregate by mass, with early studies identifying ~25% sulphur as optimal before excess binder reduces strength. Modern practice uses the minimum sulphur required to fill voids. Chempruf sulphur concrete employs 22% modified sulphur and 78% aggregate, achieving ~40 MPa strength with negligible water absorption [60]. A high-strength mix using ~20% sulphur, 5% fly ash, and basalt aggregate exceeded 70 MPa [61]. In sulphur-extended asphalt, 20–40% of bitumen is replaced with sulphur, e.g., 3.0% bitumen + 2.0% sulphur in a 5.0% binder mix [31]. Table 1 shows a summary of sulphur-based mix designs and their details.

Sulphur concrete requires no moist curing and gains strength rapidly, with demoulding in 30–60 min and full strength typically within 24 h, enabling fast precast production cycles [29,65]. Sulphur-extended asphalt behaves like hot-mix asphalt, with slightly extended workability and readiness for traffic within hours, requiring no additional curing [41]. Sulphur concrete production integrates concrete and asphalt technologies, using dried and heated aggregates, heated mixers, and temperature-controlled handling to ensure uniform coating and rapid placement [29]. Aggregates are typically heated to 150–160 °C, and mixing is completed within minutes using pugmill or rotary mixers designed for corrosion resistance [11,37]. Pre-heated moulds are required due to rapid setting, with casting similar to asphalt placement [29,64]. Recent advances include the 3D printing of sulphur concrete with precise thermal control [63,66]. Sulphur-extended asphalt can be produced in conventional plants with minor modifications, maintaining standard compaction criteria and performance [33,41].

Quality control for sulphur concrete includes ensuring dry aggregates, verifying mixing temperature (~135 °C), and assessing fresh mix flow, with adjustments made by heating, sulphur addition, cooling, or filler use [66]. Hardened properties such as compressive strength, density, and water absorption are tested early, as strength develops rapidly [29,37]. Sulphur asphalt is evaluated using standard asphalt tests, while H_2_S monitoring confirms that emissions remain within safe limits (<5 ppm) under controlled conditions [33,40,41,42,43,44,45,46,47,48,67].

### 3.2. Strength and Durability Properties

Sulphur concrete (SC) exhibits high compressive strength comparable to or exceeding conventional Portland cement concrete, with typical values of 40–60 MPa and optimised mixes reaching ~80 MPa. Rapid strength development is notable, with basalt-based SC achieving 58 MPa in 2 days compared to ~30 MPa for cement concrete at 28 days [34,68]. SC also shows a high elastic modulus (>25 GPa) and similar density to conventional concrete, though its flexural and tensile strengths are relatively lower, indicating more brittle behaviour due to the homogeneous sulphur matrix and absence of creep-related microcracking [19,37,55,61,62,69]. Polymer modification or fibre reinforcement can improve ductility, with polymers being more practical at high mixing temperatures [70]. At elevated temperatures, unmodified SC softens above ~80 °C and has limited fire resistance, although it does not propagate flames or smoke [30,31,32]. Modified sulphur can raise the softening point to 100–120 °C. At low temperatures, SC becomes stiffer but retains excellent freeze–thaw resistance due to minimal water absorption, with strength increases reported at −20 °C [17].

SC demonstrates exceptional durability in aggressive environments, showing negligible degradation in acids and salts, strong resistance to sulphate attack, and proven long-term performance in sewer and marine applications [11,29,58,60,65]. It also exhibits negligible creep and zero drying shrinkage, with thermal contraction as the primary strain mechanism [11,33,61]. Sulphur-extended asphalt improves stiffness, rutting resistance, and aging performance, with moderate sulphur contents enhancing Marshall stability and durability, particularly in hot climates [3,26,44,45,46,48,71,72,73,74]. Sulphur-based concrete and asphalt are high-performance, durable materials with rapid strength gain and exceptional chemical resistance, particularly suited to aggressive and water-limited environments. However, their broader adoption depends on optimised mix design, temperature control, and modifier selection to balance brittleness and thermal sensitivity while maximising long-term performance and sustainability benefits.

### 3.3. Performance of Sulphur Concrete vs. Conventional Concrete

The results from numerous studies confirm that sulphur concrete can achieve mechanical performance on par with conventional concrete in compression, while vastly outperforming it in certain durability aspects. Table 2 summarises the key physical and durability properties of sulphur concrete compared to conventional Portland cement concrete, highlighting the benefits of sulphur-based material. As shown, sulphur concrete excels in rapid strength development, acid resistance, impermeability, and recyclability, while its main weaknesses lie in its behaviour at high temperatures and lower ductility.

### 3.4. Applications and Case Studies

Sulphur-based construction materials have been applied or proposed in a variety of contexts, exploiting their unique properties. Table 3 categorises major application areas. Sulphur concrete and sulphur-modified asphalts find their niche in extreme conditions, such as chemical extremity (acid, salt) and environmental (freezing, space vacuum). The Bemo Rail sulphur concrete rail track sleepers eliminate the need for impregnated timber prone to rots and periodic replacement and avoid steel rebar, which can corrode in moist environments. These sleepers have passed European railway tests, demonstrating equal load-bearing capacity and vibration performance as per the standard. Additionally, after their service life, they can be melted and recast, implementing a circular economy in railway infrastructure for the first time [29].

Another successful application is sulphur concrete blocks for construction. In Kuwait, a project produced small precast sulphur concrete units for buildings and pavements, finding them simple to manufacture and with “very interesting characteristics”. These blocks were used in a trial building that showcased the material’s viability. The blocks had sharp edges and smooth surfaces, indicating good mould reproduction, and they were completely cured within hours. Their acid resistance also made them suitable for use in foundation courses where sulphate-rich soils would attack normal blocks.

### 3.5. Sustainability and Frontiers

From a sustainable development perspective, the utilisation of sulphur by-products in construction exemplifies the conversion of industrial waste into a valuable resource. Global sulphur production exceeds tens of millions of tonnes annually (approximately 70 million tonnes in the 2020s [1]), and a significant portion is not absorbed by conventional markets such as sulphuric acid manufacture. Stockpiled sulphur poses environmental risks, including dust emissions and acid runoff, while occupying valuable land. Sulphur concrete provides a long-term, stable sink for this material, effectively immobilising sulphur for decades, with the additional advantage of full recyclability at end of life. In contrast, Portland cement production accounts for nearly 8% of global CO_2_ emissions and offers limited material recovery once incorporated into concrete. Even the partial substitution of cement with sulphur-based binders could therefore yield substantial reductions in both greenhouse gas emissions and industrial waste. Life-cycle assessments suggest that sulphur-based concrete can reduce environmental impacts by up to 40% in selected applications [58]. Nevertheless, comprehensive, system-level LCA studies are still required to fully capture circularity, long-term performance, and sustainability trade-offs.

The extraterrestrial application is particularly intriguing and has reinvigorated research interest. As Wang and Snoeck [28] describe, sulphur is likely abundant on Moon/Mars in forms that can be extracted and melted. Waterless construction is a priority for off-world habitats, making sulphur concrete a leading candidate material for on a lunar base. Gruber et al. [78] recently performed the material characterisation of Martian regolith sulphur concrete and even simulated the thermomechanical loads on a Mars habitat dome made of sulphur concrete. Their experiments found that the material withstood cyclic day–night temperature swings (−60 °C to +20 °C) and remained intact under expected internal pressurisation and gravity loads.

### 3.6. Limitations and Challenges

The principal limitation is thermal sensitivity, particularly concerns over strength loss under fire exposure, which has confined its use to non-structural or compression-dominated applications such as pipes, blocks, and underground and marine works. Although fire-protective systems could enable structural use, they increase cost and complexity. Adoption is also influenced by the regional availability and price volatility of elemental sulphur, which is abundant and low-cost in some regions but requires importation in others. Health and safety considerations also play a key role. Working with hot molten sulphur requires training and precautions against burns and toxic gases. Construction crews are familiar with hot asphalt, so sulphur concrete is not entirely new; however, any mishap (overheating sulphur above ~200 °C) could release dangerous H_2_S. This has perhaps made some companies hesitant in adaptation. However, with proper automated equipment and ventilation (as used in industrial precast plants), these risks are manageable. Notably, the UAE researchers El Gamal et al. [37] developed an integrated mixing machine for sulphur concrete with safety features and demonstrated that lab-scale production can be done reliably. Most safety concerns associated with sulphur concrete production have now been effectively addressed through improved equipment design and process control. A notable industrial-scale milestone is the successful deployment of sulphur concrete railway sleepers by Bemo Rail [29], demonstrating safe and reliable manufacturing under real operating conditions. These advances suggest that further technological refinements are likely to continue mitigating production-related safety concerns and support broader industrial adoption.

## 4. Bibliometric Overview of Sulphur Application in Civil Engineering

**Figure 3:**
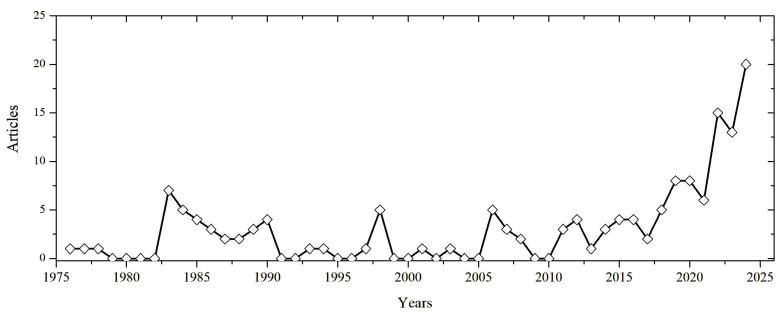
Annual publication trend in sulphur-based construction material (1970s–2025), showing a marked increase in output in the last two decades.

**Figure 4:**
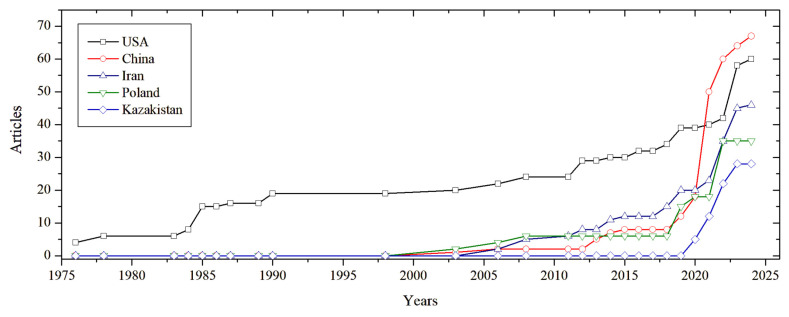
Country-wise publication over time in sulphur-based construction material (1970s–2025).

To contextualise the development of sulphur-based construction materials, a bibliometric analysis [52] was conducted on the scientific literature from 1970 to 2025. Figure 3 illustrates the annual number of publications on sulphur concrete and related topics over recent decades. Early research activity peaked in the late 1970s and 1980s following the oil embargo era (when interest in alternative materials grew). A noticeable resurgence occurred from the mid-2000s onwards, coinciding with heightened environmental focus on waste reutilisation and sustainable materials. This growth has been spurred by global sulphur oversupply and the drive for low-CO_2_ construction solutions [1]. Countries’ performance in publishing research on sulphur-based construction materials has also remarkably increased, with a larger number of effective studies reported by China and the USA (Figure 4).

**Figure 5:**
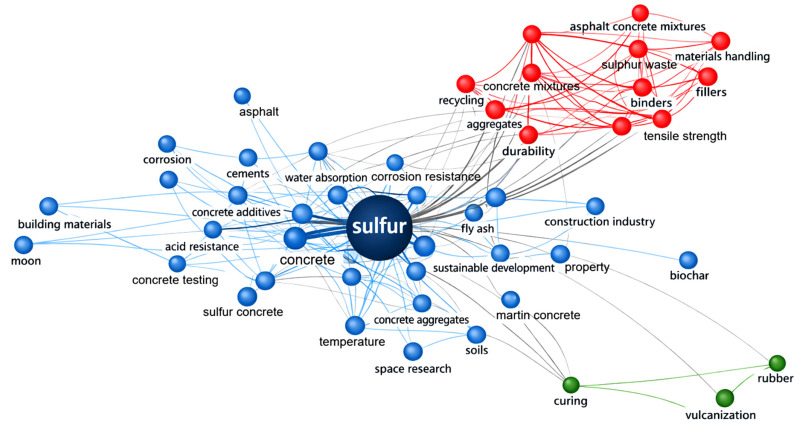
Keyword co-occurrence network showing major research clusters related to sulphur-based construction materials, including sulphur concrete, sulphur-extended asphalt, and sulphur–polymer interactions.

The co-occurrence network shown in Figure 5 provides a comprehensive visualisation of the conceptual landscape within the literature on sulphur-based construction materials. The central prominence of the term “sulphur” reflects its relevance to themes such as concretes, durability, corrosion resistance, mechanical properties, and cement replacement. The dominant blue cluster highlights long-standing research on sulphur concrete, encompassing topics such as acid resistance, water absorption, concrete additives, and emerging applications including lunar and Martian construction, demonstrating the material’s versatility and relevance to both terrestrial and extraterrestrial engineering [1,5,37,60,61,62,63,64]. The red cluster represents studies on sulphur-extended asphalt, characterised by connections to aggregates, fillers, mixture design, recycling, and material handling, indicating sustained interest in performance enhancement and sustainable pavement technologies [3,26,31,45,46]. A smaller green cluster links sulphur research to vulcanisation and rubber–sulphur chemistry, illustrating multidisciplinary intersections with polymer science. Overall, the network reveals a mature and evolving research domain with strong emphasis on performance, sustainability, and innovative applications.

**Figure 6:**
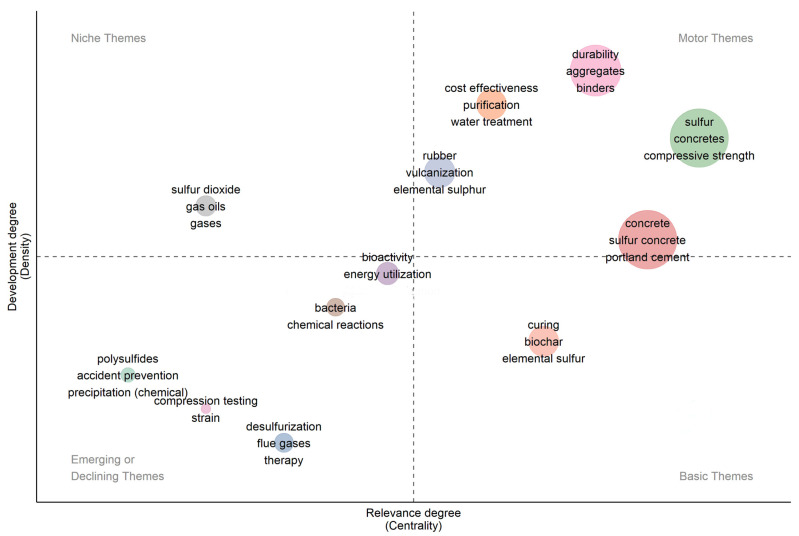
Thematic map showing motor, basic, niche, and emerging themes in sulphur-based construction materials research based on centrality and density.

The thematic map presented in Figure 6 illustrates the conceptual structure of research on sulphur-based construction materials through the dimensions of centrality (relevance) and density (development). Themes in the upper-right quadrant represent motor themes, indicating well-developed and highly influential areas such as durability, aggregates, binders, compressive strength, and sulphur concretes. These topics form the methodological and performance-oriented core of current sulphur material research. The lower-right quadrant contains basic themes, including concrete, sulphur concrete, and Portland cement, which exhibit high relevance but moderate development, reflecting foundational topics that support most studies in this field. The upper-left quadrant identifies niche themes, such as sulphur dioxide and gas oils, which are highly specialised but less central to mainstream civil engineering applications. The lower-left quadrant contains emerging or declining themes, such as desulphurisation, compression testing, and polysulfides, suggesting early-stage exploration or reduced recent emphasis. Overall, the map highlights the emergence of sulphur-based construction materials and the increasing importance of durability and composite performance.

Overall, the bibliometric overview confirms a rapidly growing and diversifying knowledge base. Initially focused on proof-of-concept and durability (1970s–1990s), research since 2000 has expanded into performance optimisation, field implementation (pavement trials), and frontier innovations (3D-printed sulphur structures for planetary habitats).

## 5. Future Prospects and Research Needs

While current results are very favourable, there remain areas for further research and development.

### 5.1. Improving Ductility and Structural Use

Finding ways to increase the tensile capacity and post-crack behaviour of sulphur concrete would open it to load-bearing structural roles. Fiber reinforcement (steel, basalt fibres) is one approach, and embedded mesh or composite action with steel sections is another. Thus, non-metallic reinforcement such as GFRP rods might be more suitable for sulphur concrete elements if needed.

### 5.2. Hybrid Systems

The concept of sulphur–polymer–cement hybrids is relatively unexplored. Imagine a concrete where a small amount of cement is included just to improve high-temperature resilience or to bond with conventional concrete elements. New chemistries like magnesium phosphate cements or geopolymers might be compatible with sulphur if carefully proportioned. So, the possibility of creating a two-phase binder that could have multi-stage hardening can also be tested.

### 5.3. Standardisation and Codes

A significant hurdle to widespread adoption is the lack of standardised design codes and specifications for sulphur concrete. Organisations like ACI have published guides [11] but no building code provisions yet. As more data becomes available on long-term performance, it would be valuable to develop codes for sulphur concrete masonry units or sulphur concrete sewer pipes, so a confident adaptation can be implemented. The circularity of sulphur concrete is also required to be studied in detail to demonstrate the effect of heating on the performance of aggregates and the resulting mechanical properties.

### 5.4. Field Demonstrations

More full-scale demonstration projects are needed to convince stakeholders. For sulphur-extended asphalt, every successful highway project builds confidence, such as the recent implementation in a Kazakhstan highway of employing sulphur asphalt, which is a big step [79]. Similar pilot projects for sulphur concrete blocks or panels in small buildings would help illustrate the practicality and identify any unforeseen issues in real environments. The Kuwait sulphur concrete block project was a good start, and others could follow in countries with sulphur surpluses [55].

### 5.5. Extraterrestrial Validation

The testing of sulphur concrete in simulated lunar/Martian conditions (vacuum, radiation, low gravity) is on the research horizon. Small prototypes in vacuum chambers, or eventually on the Moon, could show how sulphur concrete behaves outside Earth [28,63,64,78]. One challenge is how sulphur’s phase change might behave in very low gravity or extreme cold; it might actually be beneficial because cooling is quick in a vacuum, locking in the structure. In addition, the absence of atmosphere means no oxidation of sulphur, which is good.

In summary, modern sulphur concretes represent a substantial advancement over the rudimentary sulphur mortars developed a century ago, evolving into engineered composite materials that effectively address earlier limitations through optimised mix design and modification strategies. In parallel, sulphur-extended asphalts have emerged as viable modifiers for contemporary pavements, offering a compelling balance of mechanical performance, economic efficiency, and environmental benefit. The rapidly expanding body of literature, as reflected in the bibliometric overview, indicates growing recognition of this potential within both academia and industry. To enable widespread commercial adoption, future efforts should prioritise enhancing ductility and structural reliability through polymer modification and compatible reinforcement systems, establishing standardised design codes supported by long-term performance data, and executing large-scale field demonstrations. Together, these priorities directly target the technical, regulatory, and confidence barriers that must be overcome to achieve meaningful market uptake within the next decade.

## 6. Conclusions

Sulphur-based concrete and asphalt materials represent a compelling paradigm shift in construction, transforming an abundant industrial by-product into a high-performance, circular construction resource. This review demonstrates that, when properly formulated and processed, sulphur concrete consistently achieves compressive strengths of 40–60 MPa within 24 h and exhibits exceptional durability in chemically aggressive and water-limited environments. Sulphur-extended asphalts similarly show enhanced stiffness and rutting resistance, with field applications confirming performance equal to or exceeding that of conventional mixtures. The rapid strength gain, chemical resistance, zero water demand, and recyclability position sulphur-based materials as particularly attractive for precast non-structural elements, corrosion-resistant industrial infrastructure, and waterless/remote/extraterrestrial construction.

Beyond these established advantages, the trajectory of current research indicates a transition from material feasibility towards system-level innovation. Active research frontiers, including polymer-modified sulphur binders, hybrid cement–sulphur systems, additive manufacturing, and extraterrestrial construction, are reshaping sulphur concrete from a niche alternative into a platform material for advanced construction technologies. Notably, developments driven by lunar and Martian construction concepts, where waterless binders are essential, are accelerating innovations in automation, thermal control, and rapid manufacturing that are equally beneficial for terrestrial infrastructure.

Despite this progress, several critical gaps remain. The long-term behaviour of sulphur concrete under elevated temperatures, fire exposure, and sustained tensile loading requires deeper investigation, alongside standardised design methods to manage brittleness. Equally pressing is the lack of harmonised international standards, limited practitioner familiarity, and the need for robust pilot-scale demonstrations that quantify life-cycle performance, safety, and economic viability. Addressing these gaps is essential for regulatory acceptance and industry confidence.

Overall, the accumulated evidence suggests that sulphur-based construction materials are approaching a tipping point. As decarbonisation pressures intensify and circular economy strategies become imperative, sulphur concrete and sulphur-extended asphalt are well positioned to evolve from experimental solutions into mainstream materials for targeted applications. By converting sulphur stockpiles from an environmental liability into durable infrastructure assets, the construction sector can advance sustainability goals while enhancing performance, speed, and resilience on Earth and potentially beyond.

## Figures and Tables

**Table 1:** Examples of sulphur-based mix designs, processing details, and achieved properties.

Reference (Year)	Mix Description (Binder–Aggregate Composition)	Processing/ Curing	Compressive Strength (MPa)	Notes on Variation in Resulting Properties
Bacon and Davis [2]	40% elemental sulphur + 60% sand (mortar)	Cast at ~120 °C; cooled to ambient	~20 MPa (est.)	Highly acid-resistant mortar; moisture/thermal instability without modifier.
McBee et al. [60]	~22% modified sulphur (with 8% DCPD) + 78% silica aggregate (dense gradation)	Preheat agg.; mix at 135 °C; slow cool in forms	~35–42 MPa	Negligible acid attack in pH 1–3 environments; water absorption <0.2%.
Fediuk et al. [5]	20% sulphur + 5% fly ash + 75% sand/gravel (optimised dense gradation)	Mix at 140 °C; ambient cool	50–55 MPa @ 28 days	Early strength ~45 MPa @ 1 day; low shrinkage; excellent freeze–thaw resistance.
Dobrosmyslov et al. [61]	Sulphur/marshalite (finely ground 98% silicon dioxide)	Mix ~150–160 °C; mould cure 1 day	~97 MPa + MPa (high-strength)	Achieved high density and strength; XRD shows stable S_8_ crystal form.
El Gamal et al. [37]	30% sulphur + 70% sand (for pipe segments); also 5% bitumen in sulphur (BMSC variant)	Lab mixing machine at 140 °C; cast in metal mould; 24 hr gradual cool	~40 MPa (SC); ~35 MPa (BMSC)	BMSC showed ~20% lower strength but improved toughness; BMSC weight loss <0.5% after 50 freeze–thaw cycles.
Khedaywi et al. [45]	0%, 20%, 40% sulphur replacing bitumen in asphalt (limestone aggregate, 5% total binder)	Asphalt mixing at 145 °C; compact Marshall specimens	–	Marshall stability increased 15–30% with sulphur; softening point +10 °C at 40%S; slight drop in low-temp ductility.
Gul et al. [31]	~30% sulphur extended asphalt (SEA)—30% of 5.5% binder replaced by sulphur (basalt agg.)	Drum mix at 155 °C; paved lab slabs	–	30% replacement did not change optimum binder content; maintained cracking resistance per Illinois flexibility index.
Rasheed and Al-Hadidy [26]	Asphalt mix with 5% sulphur filler (substituting mineral filler in mix)	Mix at 160 °C; compaction per ASTM D6926	–	Sulphur filler (4–6% of aggregate) met ASTM mix criteria; TSR > 80%, stability > 8 kN. Recommended 5% SW filler for paving.
Sakanov et al. [46]	Two types of modified sulphur pellets (with additive) used at 30% binder replacement	Plant mixing trial; laid test sections	–	~5% cost savings vs. conventional mix; similar Marshall stability (~12 kN) and moisture resistance.
Alkhaldi and Mourad [62]	Sulphur concrete with biomass ash and industrial waste additives (to improve strength)	Lab mix ~135 °C; cast prisms	~45 MPa (with additives)	Reported improved mechanical strength and thermal stability vs. plain SC (per abstract).
Boldini et al. [63]	3D-printed sulphur concrete (70% regolith simulant, 30% sulphur) for planetary construction	Print nozzle ~140 °C; layer-by-layer deposition; controlled cooling	~20–30 MPa (est.)	Demonstrated automated construction feasibility; model predicted internal cooling rates for defect-free printing.
Munoz [3]	Sulphur extended asphalt trial—sulphur added to hot mix asphalt for roadway	Field trial (FHWA) in pavement	–	Found improved stiffness and reduced rutting; recommended further evaluation (early SEAM concept).
Giwa et al. [64]	3D printed sulphur-regolith concrete modified with dicyclopentadiene	Gaining 85% strength in 12 h	-	Minimised the sublimation rate of printed specimens in vacuum and at elevated temperature.

**Table 2:** Comparative properties of sulphur concrete (with polymer modifier) vs. ordinary Portland cement concrete [5,11,12,13,14,17,19,20,23,27,28,29,30,32,33,34,35,36,39,55,56,58,60,61,62,65,66,69,70].

Property	Sulphur Concrete (Polymer-Modified)	Portland Cement Concrete
Compressive Strength	40–60 MPa typical (achieved in 1–2 days), can reach >70 MPa with high-strength mix.	20–50 MPa (28 days curing), higher grades require special mixes, slow strength gain (70% in 7 days, 90% in 28 days).
Early Strength	~30–40 MPa within hours of casting (rapid set on cooling).	<5 MPa in first 24 h (normal concrete), requires curing, susceptible to early-age cracking.
Flexural/Tensile Strength	~10 MPa flexural, ~5 MPa tensile for 50 MPa SC (flexural: compressive, 1:6). Brittle failure (no yield).	~5 MPa flexural, ~3 MPa tensile for 40 MPa concrete (flexural: compressive, 1:9). More gradual failure (microcracking).
Modulus of Elasticity	20–30 GPa (relatively high stiffness).	25–35 GPa (normal weight concrete).
Density	~2300 kg/m^3^ (similar to normal concrete).	~2300 kg/m^3^ (normal weight).
Thermal Expansion	~α = 7–8 × 10^−6^/°C (similar to concrete).	α = 8–12 × 10^−6^/°C.
Thermal Conductivity	Lower than concrete (≈0.5–0.6 W/mK)—good insulation.	~1.6–1.8 W/mK (for dry concrete).
Service Temperature Range	~−50 °C to +80 °C (above ~80 °C, sulphur softens). Not for use in fires >120 °C without protection.	−50 °C to +300 °C (concrete can handle higher heat, though degrades >300 °C and spalls in fire if not protected).
Fire Resistance	Does not support flame (zero flame spread), but loses strength when heated; emits SO_2_ at high temp. Needs fireproof cladding for structural use in buildings.	Non-combustible, retains strength until ~500 °C; can spall explosively under rapid heating (moisture). Concrete contributes to fire resistance of structures.
Water Absorption/Permeability	Very low—<0.5% absorption; virtually impermeable (dense, no capillary pores).	5–10% absorption typical; permeable unless special mixes used. Requires water curing to reduce porosity.
Chemical Resistance (Acids, Salts)	Excellent—inert to most acids (pH 0–12), salts, sewage. No sulphate attack. Suitable for acid tanks, sewer pipes, fertiliser plants.	Poor in acids—dissolves in pH < 4 (unless special acid-resistant cement used). Vulnerable to sulphate attack, chloride penetration (corrosion).
Alkali–aggregate Reaction	Not applicable (no Portlandite or alkalis).	Potential ASR and other deleterious reactions unless aggregates/tests carefully managed.
Freeze–Thaw Durability	High, negligible internal water; durable under freeze-thaw (provided not structurally overloaded at low T). BMSC with micro-voids shows no damage 300+ cycles.	Requires air-entrainment to survive freeze-thaw; moderate scaling and internal cracking if saturated. Needs ongoing saturation protection or admixtures.
Creep and Shrinkage	Very low drying shrinkage (none, as no water loss); thermal shrinkage only on cooling. Creep also low—rigid crystal matrix.	Significant drying shrinkage (100–800 microstrain) unless controlled; creep can be substantial under load (0.2–0.3% strain in 6 months for typical stress).
Circularity	100% circular—can be reheated and recast infinitely with no strength loss. All components (sulphur and aggregate) are recoverable.	Down-cyclable—old concrete can be crushed for aggregate, but cement matrix not recoverable. Not truly reversible; reprocessing is energy-intensive and quality of recycled aggregate is lower.
Environmental Impact	Uses industrial waste sulphur (diverts from stockpiles); no CO_2_ from binder (physical process). Low CO_2_—~40% less emissions vs. OPC concrete for equivalent strength. No water needed (saves resource).	High CO_2_ footprint from cement (~0.8 t CO_2_ per 1 t cement). Significant water consumption for curing and mixing. Can incorporate other industrial by-products (fly ash, slag) to mitigate footprint.
Cost Considerations	Sulphur often cheaper than cement in regions with excess (sometimes essentially free as waste). Requires heating (energy cost) but short curing time speeds production. Specialised handling for H_2_S safety adds some cost. Overall cost can be competitive or lower, especially for precast products and pavements.	Cement cost can be significant; however, mature industry means concrete is generally economical. Additional costs for special cements or coatings in acid environments. Longer curing/turnaround times in production.

**Table 3:** Notable applications of sulphur concrete and sulphur asphalt in construction.

Application Area	Examples and Details	Rationale for Using Sulphur Material
Industrial and Chemical Infrastructure	Acid-resistant floors and sumps: Sulphur concrete used for lining pits in fertiliser plants and mining facilities. Sewer pipes and manholes: Precast sulphur concrete sewer pipes resist biogenic acid corrosion [75]. Containment vessels: Sulphur concrete tanks for acidic waste, electrolytic cells, etc., have been tested [12,13].	Exceptional chemical durability (can withstand pH 0–1, sulphates, chlorides), far outlasts OPC in acidic or saline environments. Rapid curing allows quick turnaround in repairs or new construction (minimal downtime). No rebar needed (avoids corrosion issues entirely in acid service).
Precast Building Components	Blocks, bricks, and slabs: Modified sulphur concrete masonry units produced with standard block machines. Sulphur blocks by Kuwait Institute for Scientific Research [55]. Railroad ties and sleepers: Bemo Rail (Netherlands) developed sulphur concrete railway sleepers and crane track slabs, which have passed 5-year field tests under heavy loads [29].	Fast strength gain accelerates manufacturing of blocks can be demoulded in minutes, enabling high throughput. Products are remeltable and reusable, end-of-life railway sleepers can be melted down and recast (true cradle-to-cradle recycling). Sulphur concrete products show low permeability, enhancing durability in outdoor exposure (sleepers less prone to water ingress, freeze-thaw or rotting as with timber). Lower CO_2_ footprint makes them attractive as “green” building materials (marketing as 40% CO_2_-reduced blocks).
Pavements and Roads	Road base layers: Sulphur polymer concrete has been tested as a road base material in permafrost areas (strength gain in sub-freezing temps) [76]. Surface asphalt: Several highways and test sections around the world (Canada, USA, China, Middle East) paved with sulphur-extended asphalt, a 1 km stretch in Qinghai, China (2009) using Shell Thiopave; a highway in Qatar (2012) with 40% sulphur binder [77]. Airfield pavements: Sulphur-extended asphalt trial at Paris-Orly airport on a taxiway showed improved rut resistance [77].	Cost savings by reducing expensive bitumen content (sulphur often cheaper, especially when surplus). Improved rutting resistance in hot climates due to higher binder stiffness. Potential to lower mixing and compaction temperatures (some sulphur additives act as flux), energy-saving in asphalt plant. Waterproof nature may reduce moisture damage (if properly designed to avoid stripping). Good for remote regions if sulphur available on-site (e.g., near gas plants), less bitumen to haul.
Hydraulic and Marine Structures	Dams and erosion control: There has been research on sulphur concrete for dam spillways and coastal erosion blocks (wave-dissipating units). Though not widely implemented, pilot blocks were made for jetty armour units that resist saltwater corrosion [69].	Absolute imperviousness and acid resistance are assets in water structures (no leaching, no alkali release). High early strength allows fast installation and reduces cofferdam times in marine works. Sulphur concrete is not affected by sulphate-rich soils or seawater, ideal for foundations or pipes in such conditions. For temporary works, structures could even be melted and removed later if needed (unique to sulphur concrete).
Offshore and Arctic	Offshore platform weights and structures: Sulphur concrete has been considered for underwater weights, because it is dense and corrosion-proof. Also proposed for sea outfall pipes carrying corrosive effluents. Arctic construction: In permafrost zones, building foundations using sulphur concrete can be beneficial since it sets without water [5].	Can be placed in sub-zero environments (no water to freeze, no curing needed). Resistant to saltwater corrosion and does not rust, advantageous for long-term contact with sea water. Maintains strength in cold, slightly stronger at −20 °C. No need to heat enclosure or use special cement for winter concreting, melt sulphur and pour, it will solidify and gain strength even at −40 °C.
Extraterrestrial Construction	Moon and Mars habitat modules: Recent research [28,64,78] suggests using sulphur (abundant in lunar/Martian regolith) as a binder to produce concrete-like blocks for habitats. NASA studies have made sulphur regolith concrete with compressive strength ~25 MPa. 3D-printed structures: Experiments by Gruber et al. [78], Giwa et al. [64] and Boldini et al. [63] printed elements from sulphur concrete, which could be done robotically on Mars where water is scarce. The concept “Mooncrete” or “Marscrete” often refers to sulphur-based concrete.	In situ resource utilisation (ISRU): Both Moon and Mars have sulphur available (e.g., sulphur in regolith or extracted from soil minerals). Using it avoids bringing heavy cement from Earth. No water required, a critical advantage in the space environment where water is extremely precious. Fast setting, structures can be built quickly by robots during short mission windows. Sulphur concrete’s vacuum and low-temperature performance is good (will not outgas significantly once solidified, and strength is retained in cold vacuum). Re-meltability allows errors to be fixed by reheating, and end-of-life structures to be repurposed.

## Data Availability

No new data were created or analyzed in this study. Data sharing is not applicable to this article.

## Supplementary Materials

The following supporting information can be downloaded at https://www.mdpi.com/article/10.3390/ma19040784/s1: Table S1: PRISMA 2020 Checklist.

## References

[1] Statista Research Department Production of Sulfur Worldwide in 2024, by Country. https://www.statista.com/statistics/1031181/sulfur-production-globally-by-country/.

[2] Bacon R.F., Davis H.S. (1921). Recent advances in the American sulfur industry. Chem. Metall. Eng..

[3] Munoz A. (1978). Use of Sulphur in Pavements.

[4] Gregor R., Hackl A., Bourne J.R. (1978). A New Approach to Sulfur Concrete. New Uses of Sulfur II.

[5] Fediuk R., Mugahed Amran Y.H., Mosaberpanah M.A., Danish A., El-Zeadani M., Klyuev S.V., Vatin N. (2020). A critical review on the properties and applications of sulfur-based concrete. Materials.

[6] Stel’makh S.A., Shcherban’ E.M., Beskopylny A.N., Mailyan L.R., Meskhi B., Shilov A.A., Evtushenko A., Chernil’nik A., El’shaeva D., Karalar M. (2023). Physical, mechanical and structural characteristics of sulfur concrete with bitumen modified sulfur and fly ash. J. Compos. Sci..

[7] Añón J.C.R. (1988). Guide for mixing and placing sulfur concrete in construction. ACI Mater. J..

[8] Añón J.C.R. (1984). Sulfur concrete: A construction alternative. Consult. Eng..

[9] Añón J.C.R. (1983). Sulphur concrete: A survey of its history, development, and uses. Concr. Plant Prod..

[10] Añón M.F., Añón A.K. (1983). Creep of sulphur–sand composite under uniaxial compression. Int. J. Cem. Compos. Lightweight Concr..

[11] (1993). Guide for Mixing Sulfur Concrete in Construction.

[12] Vroom A.H. (1998). Sulfur concrete for precast products. Concr. Int..

[13] Vroom A.H. (1998). Sulfur concrete goes global. Concr. Int..

[14] Añón G.T., Añón S., Añón N.S., Añón K. (1987). Studies on corrosion resistance of sulphur concrete. Key Eng. Mater..

[15] Añón S.S. (1985). Sulfur concrete for acid resistance. Chem. Eng..

[16] Añón W.C., Añón T.A., Añón B.W. (1985). Industrial evaluation of sulfur concrete in corrosive environments. Min. Eng..

[17] Añón M.D. (1987). Damage mechanism of cyclic freezing–thawing in sulfur concrete. Cem. Concr. Res..

[18] Vlahović M.M., Martinović S.P., Boljanac T.D., Jovanić P.B., Volkov-Husovic T.D. (2011). Durability of sulfur concrete in various aggressive environments. Constr. Build. Mater..

[19] Al-Tayyib A.J., Khan M.S. (1988). Evaluation of corrosion resistance of reinforcing steel in sulfur concrete. Int. J. Hous. Sci..

[20] Sabour M.R., Dezvareh G.A., Bazzazzadeh R. (2019). Corrosion prediction using the weight-loss model in sewer pipes made from sulfur and cement concretes and response surface methodology (RSM). Constr. Build. Mater..

[21] Sabour M.R., Dezvareh G.A., Pourrostami Niavol K.P. (2021). Application of artificial intelligence in modeling corrosion of cement and sulfur concrete in sewer systems. Environ. Process..

[22] Shamim Khan M., Al-Tayyib A.J. (1990). Long-term corrosion resistance of reinforcing steel in sulfur concrete. ACI Mater. J..

[23] Añón R.N., Añón T.A.R. (1986). Stability of sulphur concrete beams with steel reinforcement. Mater. Constr..

[24] Al-Hadidy A.I. (2021). Sustainable recycling of sulfur waste through utilization in asphalt paving applications. Int. J. Pavement Res. Technol..

[25] Nandal M., Sood H., Gupta P.K. (2023). A review study on sustainable utilization of waste in bituminous layers of flexible pavement. Case Stud. Constr. Mater..

[26] Rasheed S.K., Al-Hadidy A.I. (2024). Evaluation of sulfur waste as sustainable mineral filler in asphalt paving mixtures. Int. J. Pavement Res. Technol..

[27] Shin M., Kim K., Gwon S.-W., Cha S. (2014). Durability of sustainable sulfur concrete with fly ash and recycled aggregate against chemical and weathering environments. Constr. Build. Mater..

[28] Wang Q., Snoeck D. (2024). To boldly go where no one has gone before: Sulfur concrete, a promising construction material fulfilling the demands for a sustainable future on celestial objects: A review. Mater. Today.

[29] Bemo Rail BV Sustainable Crane and Railway Sleepers—100% Circular Sleepers. https://bemorail.com/railtechnology/sulphur-concrete-sleepers-2/.

[30] Ghasemi S., Nikudel M.R., Zalooli A., Khamehchiyan M., Alizadeh A., Yousefvand F., Raeis Ghasemi A.M. (2022). Durability assessment of sulfur concrete and Portland concrete under marine and lab conditions. J. Mater. Civ. Eng..

[31] Gul M.A., Khan K., Islam M.K., Shalabi F., Ozer H., Hajj R., Bhasin A. (2021). Evaluation of various factors affecting mix design of sulfur-extended asphalt mixes. Constr. Build. Mater..

[32] Medvedeva G.A., Akhmetova R.T., Labutkin A.G. (2016). Use of wastes from thermal power industry in manufacturing of high-strength sulfur concrete. Res. J. Pharm. Biol. Chem. Sci..

[33] Shell (2010). Shell Thiocrete—Technologies for Sulphur-Enhanced Concrete.

[34] Czarnecki B., Gillott J.E. (1990). Effect of admixtures on durability of sulphur concrete made with various aggregates. Eng. Geol..

[35] Czarnecki B., Gillott J.E. (1989). Effect of admixtures on strength of sulphur concrete. Cem. Concr. Aggreg..

[36] Czarnecki B., Gillott J.E. (1990). Effect of mix design on sulfur concrete properties. Cem. Concr. Aggreg..

[37] El Gamal M., El-Sawy K., Mohamed A.-M.O. (2021). Integrated mixing machine for sulfur concrete production. Case Stud. Constr. Mater..

[38] Syroezhko A.M., Begak O.Y., Fedorov V.V., Gusarova E.N. (2003). Modification of paving asphalts with sulfur. Russ. J. Appl. Chem..

[39] Abdel-Jawad Y., Al-Qudah M. (1994). Combined effect of water and temperature on sulfur concrete strength. Cem. Concr. Res..

[40] Alghrafy Y.A., El-Badawy S.M., Abd Alla E.M. (2021). Rheological and environmental evaluation of sulfur-extended asphalt binders modified by high- and low-density polyethylene recycled waste. Constr. Build. Mater..

[41] Bieliatynskyi A., Yang S., Krayushkina K., Shao M., Ta M. (2023). Study of the possibility of using sulfur asphalt and sulfur concrete in road construction. Mater. Sci.-Pol..

[42] Chen J.S., Huang C.C. (2007). Fundamental characterization of SBS-modified asphalt mixed with sulfur. J. Appl. Polym. Sci..

[43] Elkholy S.A., Abd El-Rahman A.M.M., El-Shafie M., Abo-Shanab Z.L. (2018). Physical and rheological properties of modified sulfur asphalt binder. Int. J. Pavement Res. Technol..

[44] Gawel I., Yen T.F., Chilingarian G.V. (2000). Sulphur-Modified Asphalts. Developments in Petroleum Science.

[45] Khedaywi T., Haddad M., Mujalli R., Shareef S. (2023). Effect of sulfur on the asphalt cement and asphalt concrete mixture: State of the art. Innov. Infrastruct. Solut..

[46] Sakanov D.K., Aspanbetov D.A., Sakanov K.T., Nurmetov S., Borshchev N.V., Lou B., Barbieri D.M. (2024). Characterization of two types of modified sulphur pellets for sulfur-extended asphalt mixtures. Results Eng..

[47] Souaya E.R., Elkholy S.A., Abd El-Rahman A.M.M., El-Shafie M., Ibrahim I.M., Abo-Shanab Z.L. (2015). Partial substitution of asphalt pavement with modified sulfur. Egypt. J. Pet..

[48] Trang N.T., Hung T.N., Khang P.H., Cậy B.X., Kien B.N. (2021). Research on the effects of sulfur on characteristics of sulfur bituminous binder (SBB) and hot mix asphalt-sulfur (HMAS). Transp. Commun. Sci. J..

[49] Linares-Espinós E., Hernández V., Domínguez-Escrig J.L., Fernández-Pello S., Hevia V., Mayor J., Padilla-Fernández B., Ribal M.J. (2018). Methodology of a systematic review. Actas Urológicas Españolas.

[50] Page M.J., McKenzie J.E., Bossuyt P.M., Boutron I., Hoffmann T.C., Mulrow C.D., Shamseer L., Tetzlaff J.M., Akl E.A., Brennan S.E. (2021). The PRISMA 2020 statement: An updated guideline for reporting systematic reviews. bmj.

[51] Mingers J., Leydesdorff L. (2015). A review of theory and practice in scientometrics. Eur. J. Oper. Res..

[52] Aria M., Cuccurullo C. (2017). bibliometrix: An R-tool for comprehensive science mapping analysis. J. Informetr..

[53] Aromataris E., Pearson A. (2014). The systematic review: An overview. AJN Am. J. Nurs..

[54] Haddaway N.R., Page M.J., Pritchard C.C., McGuinness L.A. (2022). PRISMA2020: An R package and Shiny app for producing PRISMA 2020-compliant flow diagrams, with interactivity for optimised digital transparency and open synthesis. Campbell Syst. Rev..

[55] Al-Otaibi S., Al-Aibani A., Al-Bahar S., Abdulsalam M., Al-Fadala S. (2019). Potential for producing concrete blocks using sulphur polymeric concrete in Kuwait. J. King Saud Univ. Eng. Sci..

[56] Shavkatova D., Turaev K., Amanova N., Go’zal R., Lal B., Beknazarov K., Elyor E., Hosseini-Bandegharaei A., Aliev N. (2024). Preparing a new type of concrete based on sulfur-melamine modifier. Baghdad Sci. J..

[57] Hashami M., Ongarbayev Y., Tileuberdi Y., Imanbayev Y., Zhambolova A., Kanzharkan Y. (2025). Technological Progress in Sulfur-Based Construction Materials: The Role of Modified Sulfur Cake in Concrete and Bitumen. Appl. Sci..

[58] Amanova N., Turaev K., Shadhar M.H., Tadjixodjayeva U., Jumaeva Z., Berdimurodov E., Eliboev I., Hosseini-Bandegharaei A. (2024). Sulfur-based concrete: Modifications, advancements, and future prospects. Constr. Build. Mater..

[59] Zhang T., Wu C., Li B., Wang J., Ravat R., Chen X., Wei J., Yu Q. (2019). Linking the SO_2_ emission of cement plants to the sulfur characteristics of their limestones: A study of 80 NSP cement lines in China. J. Clean. Prod..

[60] McBee W.C., Sulliven T.A., Jong B.W. Modified Sulfur Concrete Technology. Proceedings of the SULFUR-81 International Conference on Sulfur.

[61] Dobrosmyslov S.S., Zadov V.E., Nazirov R.A., Nagibin G.E., Voronin A.S., Simunin M.M., Fadeev Y.V., Khartov S.V. (2022). High strength construction material based on sulfur binder obtained by physical modification. Buildings.

[62] Al Khaldi V., Mourad A.H. (2025). Black magic for concrete: Using biomass and industrial by-products to create innovative sustainable construction materials. Case Stud. Constr. Mater..

[63] Boldini A., Giwa I., Kamel E., Kazemian A. (2025). Spatiotemporal temperature prediction in 3D-printed sulfur concrete for automated construction on Earth and beyond. Autom. Constr..

[64] Giwa I., Dempsey M., Fiske M., Kazemian A. (2024). 3D printed sulfur-regolith concrete performance evaluation for waterless extraterrestrial robotic construction. Autom. Constr..

[65] Mohamed A.-M.O., El-Gamal M. (2010). Sulfur Concrete for the Construction Industry: A Sustainable Development Approach.

[66] Yeoh D., Koh K.H., Jamaluddin N. (2015). Exploratory study on the mechanical and physical properties of concrete containing sulfur. J. Teknol..

[67] Faramarzi M., Golestani B., Lee K.W. (2017). Improving moisture sensitivity and mechanical properties of sulfur extended asphalt mixture by nano-antistripping agent. Constr. Build. Mater..

[68] Tunc E.T. (2018). Effects of basalt aggregates on concrete properties. Qual. Stud..

[69] Khademi A.G., Sar H.I.K. (2015). Comparison of sulfur concrete, cement concrete and cement-sulfur concrete and their properties and application. Curr. World Environ..

[70] Chashm Khavari B., Shekarriz M., Aminnejad B., Lork A., Vahdani S. (2023). Laboratory evaluation and optimization of mechanical properties of sulfur concrete reinforced with micro and macro steel fibers. Constr. Build. Mater..

[71] Shi K., Ma F., Fu Z., Lou B., Barbieri D.M., Li J., Song R., Yuan D. (2025). Comprehensive evaluation of sulfur content and curing duration effects on the rheological performance of sulfur-extended bitumen. Fuel.

[72] Hasan N.H., Al-Hadidy A.I. (2025). Comparative Performance of Carbon-Sulfur Asphalt Mixtures Containing ABS and SBS Polymers Under Short and Long-Term Aging Impact. Int. J. Pavement Res. Technol..

[73] Duan K., Wang C., Liu J., Song L., Chen Q., Chen Y. (2022). Research progress and performance evaluation of crumb-rubber-modified asphalts and their mixtures. Constr. Build. Mater..

[74] Gulzar M.A., Rahim A., Ali B., Khan A.H. (2021). An investigation on recycling potential of sulfur concrete. J. Build. Eng..

[75] (2000). Sulfur Concrete for Corrosion-Resistant Sewer Pipe, Concrete Pipe for the New Millennium.

[76] Cowe Falls L.G., Haas R. (1980). Pavement Design for Permafrost Conditions: Structural and Thermal Requirements.

[77] Ullah A., Wen H.P., Ullah Z., Ali B., Khan D. (2024). Evaluation of high modulus asphalts in China, France, and USA for durable road infrastructure, a theoretical approach. Constr. Build. Mater..

[78] Gruber S.H., Bode M., Marcher T., Lackner R. (2025). Thermomechanical loading scenarios of habitat structures on Mars: Experimental material characterization and numerical assessment of sulfur-concrete constructions. Dev. Built Environ..

[79] Nugmanova A.U., Yelshibayev A.O. (2023). Enhancing Road Infrastructure in Kazakhstan with Modified Sulfur and Low-Strength Inert Materials. Smart Geotechnics for Smart Societies.

